# ELMO1 Regulates RANKL-Stimulated Differentiation and Bone Resorption of Osteoclasts

**DOI:** 10.3389/fcell.2021.702916

**Published:** 2021-07-26

**Authors:** Xinyue Liang, Yafei Hou, Lijuan Han, Shuxiang Yu, Yunyun Zhang, Xiumei Cao, Jianshe Yan

**Affiliations:** ^1^School of Life Sciences, Shanghai University, Shanghai, China; ^2^Department of Immunology and Microbiology, Shanghai Institute of Immunology, Shanghai Jiao Tong University School of Medicine, Shanghai, China

**Keywords:** ELMO1, osteoclast differentiation, bone resorption, adhesion, migration

## Abstract

Bone homeostasis is a metabolic balance between the new bone formation by osteoblasts and old bone resorption by osteoclasts. Excessive osteoclastic bone resorption results in low bone mass, which is the major cause of bone diseases such as rheumatoid arthritis. Small GTPases Rac1 is a key regulator of osteoclast differentiation, but its exact mechanism is not fully understood. ELMO and DOCK proteins form complexes that function as guanine nucleotide exchange factors for Rac activation. Here, we report that ELMO1 plays an important role in differentiation and bone resorption of osteoclasts. Osteoclast precursors derived from bone marrow monocytes (BMMs) of Elmo1^–/–^ mice display defective adhesion and migration during differentiation. The cells also have a reduced activation of Rac1, p38, JNK, and AKT in response to RANKL stimulation. Importantly, we show that bone erosion is alleviated in Elmo1^–/–^ mice in a rheumatoid arthritis mouse model. Taken together, our results suggest that ELMO1, as a regulator of Rac1, regulates osteoclast differentiation and bone resorption both *in vitro* and *in vivo*.

## Introduction

Bone homeostasis is a metabolic balance between the formation and resorption of the bone in which osteoblasts and osteoclasts play crucial roles, respectively. When bone resorption goes beyond formation, the homeostasis is disrupted with the consequence of low bone mass and causing bone diseases such as rheumatoid arthritis (RA), which is characterized by the presence of inflammatory synovitis accompanied by bone destruction mainly caused by osteoclasts. Derived from BMM lineage cells, osteoclast precursors differentiate into osteoclasts under the regulation of two cytokines, namely, macrophage colony-stimulating factor (M-CSF) and Receptor activator of NF-kB ligand (RANKL). Binding of M-CSF to its receptor c-Fms leads to the activation of downstream signal pathways such as AKT and mitogen-activated protein kinases (MAPKs) including extracellular signal-regulated kinase (ERK), c-Jun N-terminal kinase (JNK), and p38 and resulting in the proliferation and survival of osteoclast precursors. Association of RANKL with its receptor RANK triggers the signal transduction pathways to induce the differentiation of osteoclast precursors to giant multinucleated osteoclasts, which play a major role in the degradation and resorption of the bone matrix (Lee and States, 2000; Lee et al., 2018).

As an essential osteoclastogenic factor, RANKL governs the signal pathways to induce the differentiation of osteoclast precursors into osteoclasts (Park et al., 2017; Supplementary Figure 1). Activation of RANK by binding to RANKL prompts the cytoplasmic recruitment of the TNF receptor-associated factor (TRAF) proteins, in which TRAF6 is found to play a major role in regulating osteoclast differentiation (Lomaga et al., 1999; Naito et al., 1999; Lamothe et al., 2007). By forming a complex with Src, TRAF6 activates downstream signal transduction pathway PI3K (Wong et al., 1999), resulting in the generation of phosphatidylinositol-(3,4,5)-trisphosphate (PIP3). This phospholipid leads to the membrane translocation of proteins containing pleckstrin homology domains, such as Cytosolic Regulator of Adenylyl Cyclase and Protein Kinase B (Akt/PKB) (Parent and Devreotes, 1999; Sasaki et al., 2004). Akt is reported to be involved in osteoclast differentiation (Lee et al., 2002). The phosphatase PTEN functions as an antagonist of PI3K by dephosphorylating PIP3 to generate PIP2 and suppresses RANKL-mediated osteoclast differentiation (Funamoto et al., 2002; Iijima and Devreotes, 2002; Sugatani et al., 2003). By forming a complex with transforming growth factor–β (TGF-β)–activated kinase 1 (TAK1) and TAK1-binding protein2 (TAB2), TRAF6 transduce the RANK signaling to activate downstream MAPKs such as p38, JNK and ERK to play vital roles in the differentiation of osteoclast precursors (David et al., 2002; Li et al., 2002; Mizukami et al., 2002; He et al., 2011). RANKL-TRAF6 signaling also activates IκB kinase (IKK), which, in turn, phosphorylates IκB for degradation that ultimately releases NF-κB from its inactive state. IKKβ is found to play a critical role for RANKL-stimulated degradation of IκB and is required for osteoclastogenesis *in vitro* and *in vivo* (Ruocco et al., 2005). Released NF-κB and activation of MAPKs eventually induce the master osteoclast transcription factor, the nuclear factor of activated T cells cytoplasmic 1 (NFATc1) (Asagiri et al., 2005), and promote the expression of related genes such as cathepsin K, osteoclast-associated receptor (OSCAR), and tartrate-resistant acid phosphatase (TRAP) during terminal differentiation of osteoclasts (Reddy et al., 1995; Matsumoto et al., 2004; Kim et al., 2005).

Rho GTPases have been found to play important roles in osteoclast differentiation and function (Gao et al., 2020). By regulating the cytoskeleton rearrangement, Rho GTPases control migration of mononuclear osteoclast precursors to form multinucleated osteoclasts, and to form the actin ring, the specific organization for bone resorption (Touaitahuata et al., 2014). Much work have demonstrated that Rac GTPase plays a pivotal role in activation of MAPKs signaling pathways (Lopez-Bergami et al., 2005; Guo et al., 2013). It has been reported that Rac1 is involved in the RANKL-dependent activation of p38 MAPK in osteoclast precursors (Lin et al., 2015), and that Dock5, p130Cas, and CrkII play essential roles in function of osteoclasts through activation of Rac1 (Vives et al., 2011; Nagai et al., 2013; Kim et al., 2016). Importantly, a recent study demonstrated that Rac1 and Cdc42 exchange factor Triple functional domain (Trio) is critical for bone resorption, and mice bearing the conditional knockout of Trio showed increased bone mass due to the impaired bone resorption (Gu et al., 2020). Amongst other pathways, Dock180 family members function in conjunction with the ELMO (Engulfment and cell motility) family proteins as a guanine nucleotide exchange factor (GEF) to activate Rac GTPase (Reddien and Horvitz, 2004). In previous studies, we have demonstrated that chemoattractant-stimulation of G-protein-coupled receptors (GPCRs) trigger downstream heterotrimeric G-proteins, such as Gα and Gβγ subunits, to form complexes with ELMO family proteins, which in turn activate Rac GTPase to regulate F-actin dynamics during migration of *D. discoideum* and cancer cells (Yan et al., 2012; Li et al., 2013; Wang et al., 2016). Recently it was reported that ELMO1, as a cytoplasmic regulator in neutrophils, is involved in inflammatory arthritis (Arandjelovic et al., 2019).

Despite much progress, our understanding on the role and regulation of signaling networks controlling osteoclast differentiation and function remains incomplete. In this study, we report the roles of ELMO1 in RANKL-induced osteoclast differentiation. Our data indicate that ELMO1 regulates the adhesion and migration of osteoclast precursors, and participates in osteoclast differentiation through Rac1, AKT, p38, and JNK signal pathways. In addition, we show that activity of bone resorption is compromised in osteoclasts lacking ELMO1, and Elmo1 knockout mice exhibit alleviated bone erosion in a serum transfer induced rheumatoid arthritis model, suggesting that ELMO/DOCK/RAC axis regulates RANKL-stimulated osteoclast differentiation and bone resorption activity, both *in vitro* and *in vivo*.

## Results

### ELMO1 Plays a Role in Bone Resorption by Affecting Osteoclast Differentiation

**FIGURE 1:**
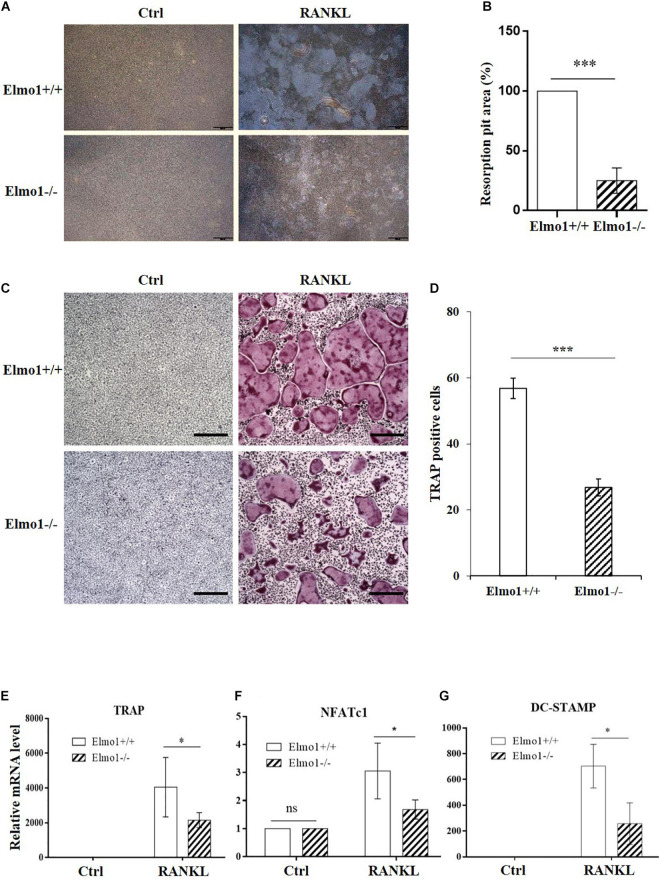
Elmo1-deficient osteoclasts show impaired bone resorption activity and differentiation. **(A)** Pit formation assay. BMMs were cultured with M-CSF alone or M-CSF and RANKL on inorganic crystalline calcium phosphate plates. Attached cells were removed and resorption lacunae were visualized by bright-field microscopy. One representative photograph is shown. Scale bar, 500 μm. **(B)** Pit areas were quantified using ImageJ and graphed. Data are indicated as means ± SEM (*n* = 3). **(C)** RANKL-induced osteoclast differentiation of BMMs. Mouse BMMs were cultured in the presence of M-CSF and RANKL for 4 days. The cells were fixed and stained for TRAP staining. One representative picture is shown. Scale bar, 100 μm. **(D)** The numbers of TRAP-positive cells were counted and graphed. Data are indicated as means ± SEM (*n* = 13). **(E–G)** Relative mRNA level of TRAP, NFATc1, and DC-STAMP. Osteoclast precursors were treated with M-CSF and RANKL for 4 days, then relative mRNA level of TRAP (*n* = 6), NFATc1 (*n* = 4), and DC-STAMP (*n* = 3) were determined by qRT-PCR. Data are indicated as means ± SEM. Statistical significance was assessed by *t*-test, *P < 0.05 and ***P < 0.001.

**FIGURE 2:**
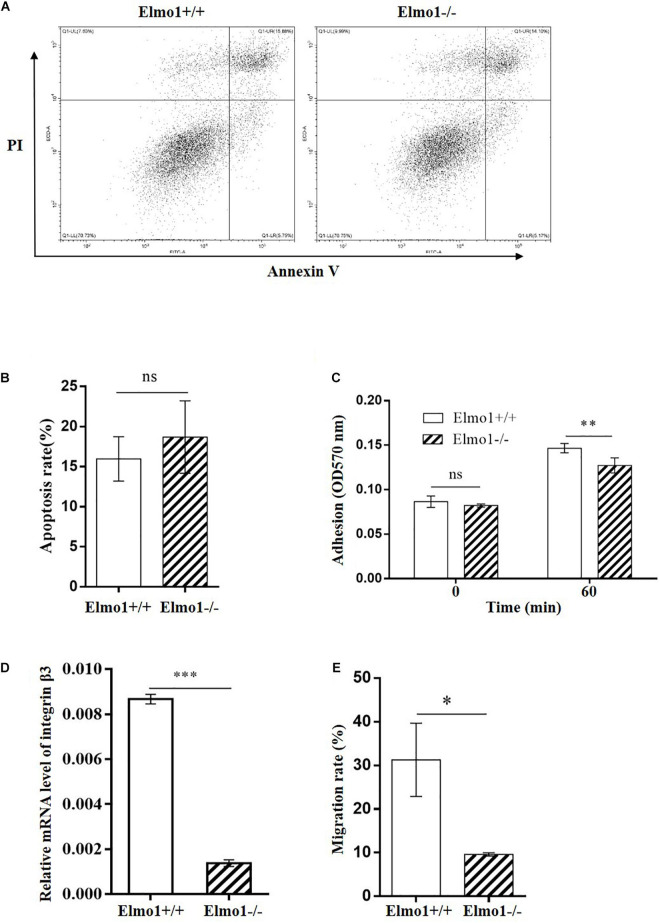
Effect of Elmo1 deficiency on apoptosis, adhesion and migration of osteoclast precursors. **(A)** Apoptosis of osteoclast precursors. RANKL-induced osteoclasts were stained with Annexin V-FITC and PI to detect the apoptosis by flow cytometry. One representative result is shown. **(B)** The percentage of Annexin V-FITC positive cells were quantified with FlowJo software and the data was indicated as means ± SEM (*n* = 4). **(C)** Osteoclast precursors adhesion assay. Cells were incubated at 37°C for 0 or 60 min, and stained with crystal violet followed by DMSO dissolving for measuring absorbance at 570 nm. The data was indicated as means ± SEM (*n* = 4). **(D)** Relative mRNA level of integrin β3 in osteoclast precursors. Cells were treated with RANKL for 4 days in the presence of M-CSF, then relative mRNA level of integrin β3 was determined by qRT-PCR. The data was indicated as means ± SEM (*n* = 3). **(E)** Osteoclast precursors migration assay. Cells were allowed to migrate under stimulation of M-CSF for 24 h. Migrated cell numbers in the bottom chamber were counted. The data was indicated as means ± SEM (*n* = 3). Statistical significance was assessed by *t*-test, *P < 0.05, **P < 0.01, ***P < 0.001, and ^*ns*^*P* > 0.05.

To investigate the impact of Elmo1 in osteoclasts, we isolated BMMs from Elmo1^+/+^ (wild-type) and Elmo1^–/–^ (knockout) mice that came from heterozygote mating (Supplementary Figure 2), and treated them with RANKL in the presence of M-CSF on inorganic crystalline calcium phosphate plates. Elmo1^–/–^ osteoclasts displayed a significant decreased bone resorption activity compared to Elmo1^+/+^ osteoclasts (Figures 1A,B). To examine whether ELMO1 is involved in osteoclast differentiation, osteoclast precursors were cultured with M-CSF and RANKL and then stained for TRAP, a marker of mature osteoclasts. We found that the formation of TRAP-positive multinucleated cells containing more than three nuclei was remarkably higher in wild-type cells compared to that in Elmo1^–/–^ cells (Figures 1C,D). One representative image of multinucleated cells with a higher magnification is shown in Supplementary Figure 3. Moreover, the mRNA level of TRAP, NFATc1, and DC-STAMP, which are essential factors for osteoclast differentiation, were much higher in wild-type cells relative to those in Elmo1^–/–^ cells (Figures 1E–G). Considering the fact that RANKL may induce cell apoptosis (Bharti et al., 2004), we next examined whether the defect of osteoclast differentiation in Elmo1^–/–^ cells was a consequence of reduced cell number caused by the treatment of RANKL. To rule out this possibility, we detected the cell apoptosis by Annexin V assay. It turned out that although RANKL treatment indeed triggered cell apoptosis, there was no significant difference between Elmo1^+/+^ and Elmo1^–/–^ cells (Figures 2A,B). Taken together, our data suggest that ELMO1 plays a role in bone resorption by affecting osteoclast differentiation.

### Elmo1 Deficiency Suppresses the Adhesion and Migration of Osteoclast Precursors

Adherence and migration to the bone surface play essential roles during early stage of osteoclast differentiation (Kim et al., 2015). Thus, we assessed the effect of ELMO1 on adhesion and migration in osteoclast precursors. We found that osteoclast precursors derived from Elmo1^–/–^ mice showed significant reduced adhesion capacity compared to the cells from wild-type mice (Figure 2C). Consistently, the expression of integrin β3, which is required for cell adhesion (Jung et al., 2012), was remarkably downregulated in the Elmo1^–/–^ osteoclast precursors (Figure 2D). In addition, we assessed the expression of other adhesion molecules such as cadherin-2 and cadherin-11. Surprisingly, the mRNA levels of them in KO cells were much higher than that in WT cells (Supplementary Figure 4). We also detect integrin β3 and cadherins by immunofluorescence assay. However, there was no significant difference in the distribution and fluorescence intensity of these molecules between the WT and KO cells (Supplementary Figures 5–7). These results suggested that ELMO1 may play diverse roles in regulating the expression of adhesion molecules. Future work is needed to reveal the detailed mechanism of this phenomenon. We then examined the migration of osteoclast precursors stimulated by M-CSF. Elmo1^–/–^ osteoclast precursors exhibited significant decreased chemotactic migration rate in a transwell assay (Figure 2E) compared to wild-type cells. These findings indicate that Elmo1 deficiency suppresses the capacities of adhesion and migration of osteoclast precursors, which are critical processes for mature osteoclasts formation.

### ELMO1 Upregulates RANKL-Induced Activation of Rac1, MAPKs, and AKT

**FIGURE 3:**
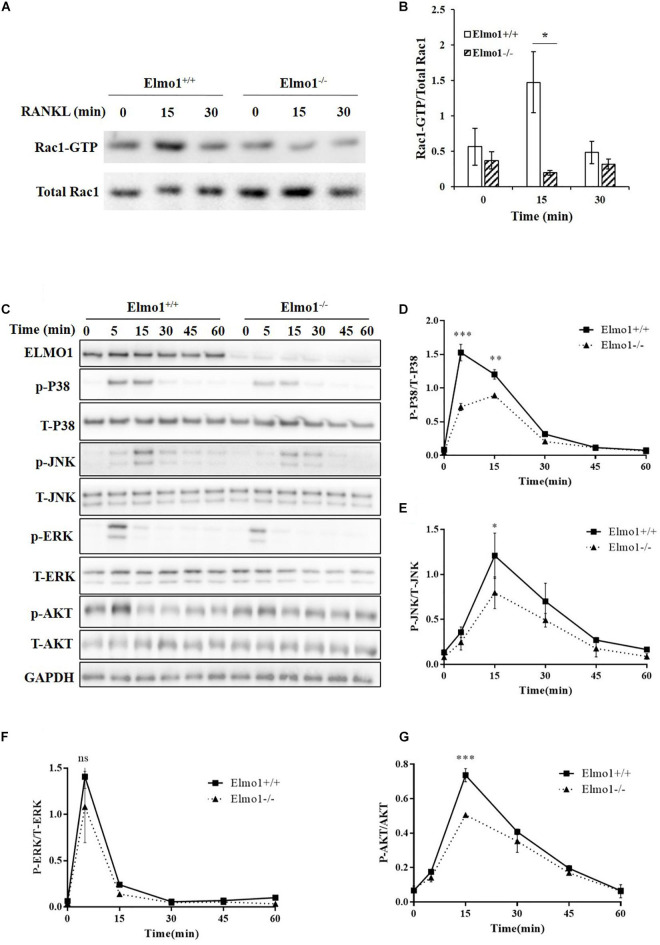
Elmo1 upregulates RANKL-induced activation of Rac1, MAPKs, and AKT. **(A)** RANKL-induced Rac1 activation assay. Osteoclast precursors were lysed after RANKL stimulation for 15 and 30 min. The lysates were incubated with PAK-PBD beads, and proteins complexed to the beads were subjected to SDS-PAGE and analyzed by immunoblotting using anti-Rac1. **(B)** The ratio of Rac1-GTP to total Rac1 was quantified and graphed. Data are indicated as means ± SEM (*n* = 3). **(C)** Osteoclast precursors were stimulated by RANKL for the indicated time. Cell lysates were subjected to SDS-PAGE and analyzed by immunoblotting for detecting phosphorylation form and total p38, JNK, ERK, and AKT. **(D–G)** The intensity of phosphorylated p38 (*n* = 3), JNK (*n* = 4), ERK (*n* = 3), and AKT (*n* = 3) was quantified by densitometry using ImageJ software and expressed as the ratio of phosphorylated form to total protein. Data are indicated as means ± SEM. Statistical significance was assessed by *t*-test, *P < 0.05, **P < 0.01, ***P < 0.001, and ^*ns*^*P* > 0.05.

Because Rac1 was found to play an important role in the differentiation of BMMs into osteoclasts through p38 activation (Lin et al., 2015), we tested whether ELMO1 regulates this process through activation of Rac1 and MAPKs. As expected, Rac1 activation was considerably impaired in Elmo1^–/–^ cells (Figures 3A,B) upon RANKL stimulation. Since ELMO1 is involved in Rac1 signaling that modulates actin reorganization, we visualized the cytoskeleton by staining F-actin with Alexa Fluor 633-phalloidin. As expected, we found that phalloidin was enriched at the actin-ring of giant multinucleated osteoclasts, particularly in WT cells, suggesting that ELMO1 may be involved in the actin-ring formation through activation of Rac1 (Supplementary Figure 8). We next examined whether ELMO1 regulates the activation of MAPKs with stimulation of RANKL. Relative to wild-type cells, Elmo1^–/–^ cells exhibited a significant reduction in the level of phosphorylation of p38 and JNK (Figures 3C–E). Phosphorylation of ERK in Elmo1^–/–^ cells was decreased to a certain degree as well although it was not significant (Figure 3F). In addition, we found that phosphorylation of AKT got reduced in Elmo1^–/–^ cells (Figure 3G). Our data suggest that ELMO1 plays a role in activation of Rac1, MAPKs, and AKT under the stimulation of RANKL in the differentiation of osteoclasts.

### Bone Erosion in Elmo1^–/–^ Mice Is Alleviated in a Rheumatoid Arthritis Model

**FIGURE 4:**
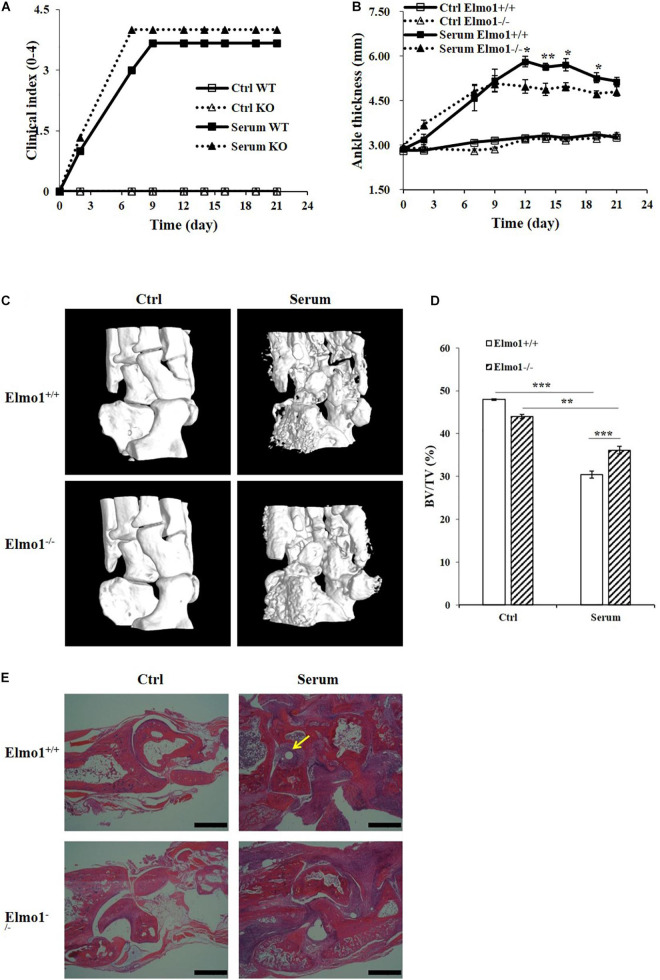
Bone erosion of Elmo1^–/–^ mice is alleviated in rheumatoid arthritis. Generation of serum transfer mouse model of arthritis was performed as mentioned in the materials and methods. **(A)** Arthritis was monitored by clinical index. Data are indicated as means ± SEM (*n* = 6). **(B)** Measurement of ankle thickness over time. Data are indicated as means ± SEM (*n* = 6). **(C)** Representative micro-CT images of ankle joints are shown. **(D)** Bone volume as a fraction of total bone volume (BV/TV) of ankle joints cortical bone. Data are indicated as means ± SEM (*n* = 6). **(E)** H&E-stained corresponding histological joint sections. Yellow arrow indicates site of bone erosion. Scale bar, 200 μm. Statistical significance was assessed by *t*-test, *P < 0.05, **P < 0.01, and ***P < 0.001.

To further investigate the importance of ELMO1 on osteoclast bone resorption function *in vivo*, we compared the focal bone erosion in mice by generating RA model which displays many of the characteristic features including bone destruction (Pettit et al., 2001). To accomplish this, Elmo1^–/–^ mice and their littermates of Elmo1^+/+^ were injected with serum from K/BxN arthritic mice, and the progression of arthritis was monitored by recording clinical index and ankle thickness over time. As expected, both Elmo1^+/+^ and Elmo1^–/–^ mice injected with K/BxN serum developed severe arthritis compared to the PBS-injected controls, manifesting the onset of clinical signs (Figure 4A) and increase of ankle thickness (Figure 4B) within 6 days. However, the ankle thickness in Elmo1^–/–^ mice was significantly lower than it in Elmo1^+/+^ mice after 12 days of serum transfer (Figure 4B), suggesting that Elmo1 deficiency ameliorates the progression of arthritis.

Moreover, micro-CT of ankle joints and histological analyses of corresponding sections were used to assess the degree of bone erosion in the mice. As shown in Figure 4C, Elmo1^–/–^ mice exhibited a marked reduction of bone erosion compared to Elmo1^+/+^ mice. We further found that the ratio of bone volume to total volume (BV/TV) and the bone mineral density (BMD) of cortical bone of the ankle joints in Elmo1^+/+^ mice was significantly lower than in Elmo1^–/–^ mice, while the ratio of bone surface to bone volume (BS/BV) in Elmo1^+/+^ mice was significantly higher than it in Elmo1^–/–^ mice (Figure 4D and Supplementary Figures 9A,B). Consistent with this data, lower degree of bone destruction was found in Elmo1^–/–^ mice in the matched H&E-stained sections (Figure 4E). The substantial reduction in bone erosion could be a consequence of decreased number of mature TRAP-stained osteoclasts in Elmo1^–/–^ mice, as shown in and Supplementary Figure 10. Taken together, our data indicated that Elmo1 deficiency reduces bone resorption in Elmo1^–/–^ mice.

## Discussion

The evidence presented here reveals the biological functions of ELMO1 in osteoclast for differentiation and bone resorption. We showed that ELMO1 modulates the differentiation of osteoclast via regulating the adhesion and migration of osteoclast precursors. We found that ELMO1 participates in RANKL-induced osteoclast differentiation through Rac1, p38, JNK, and AKT signal pathways. Furthermore, we discovered that bone erosion is alleviated in Elmo1 knockout mice in rheumatoid arthritis model. Taken together, our data suggest that ELMO1 serves as a regulator for the RANKL-induced osteoclast differentiation and bone resorption.

Previous studies in *C. elegans, D. discoideum*, cancer cells, and neutrophils have established that ELMO family proteins play a crucial role in cell migration (Reddien and Horvitz, 2004; Côté and Vuori, 2007; Yan et al., 2012; Li et al., 2013; Wang et al., 2016; Arandjelovic et al., 2019). In this study, we showed that deletion of Elmo1 caused defective migration in osteoclast precursors as well (Figure 2E). Our data supports the notion that ELMO/DOCK complex functions as an evolutionarily conserved GEF to regulate Rac activation in cell migration. We found that osteoclasts lacking ELMO1 had a lower bone-resorbing activity *in vitro* (Figures 1A,B), which was associated with abnormal phenotypes characterized by the less multinucleated cells formed when BMMs were developed with the treatment of RANKL (Figures 1C,D). In line with our findings, Lin and colleagues reported that RANKL-stimulated osteoclast formation in Rac1-overexpressing cells is significantly enhanced (Lin et al., 2015). In addition, it was found that osteoclasts lacking Dock5, the Rac1 exchange factor, have impaired adhesion (Vives et al., 2011). We therefore propose that ELMO1 and Rac1 function in regulating RANKL-induced osteoclast differentiation during bone resorption.

It has been well established that RANK recruits TRAF6 to form a TRAF6-TAB2-TAK1 complex, which in turn activates downstream MAPKs to regulate the development and function of osteoclasts (Mizukami et al., 2002; Lee et al., 2016). Rac1 is known to activate numerous signal pathways such as JNK and p38 (Van Aelst and D’Souza-Schorey, 1997; Etienne-Manneville and Hall, 2002). We therefore, examined whether ELMO1, as the upstream regulator for Rac1, was involved in these RANKL-stimulated signal pathways. Our results showed that RANKL-triggered phosphorylation of p38 and JNK occurred but with a significantly lower level in Elmo1^–/–^ osteoclast precursors compared to Elmo1^+/+^ cells, indicating that ELMO1 functions in RANKL-mediated activation of p38 and JNK (Figures 3C–E). A recent study revealed a novel role for ELMO1 which functions as a cytoplasmic regulator downstream of neutrophil receptors for C5a and LTB4 and thereby promotes inflammatory arthritis (Arandjelovic et al., 2019). Interestingly, we also demonstrated in this study that bone erosion in Elmo1^–/–^ mice is alleviated in a K/BxN serum transfer induced rheumatoid arthritis model, indicating that ELMO1 also affects bone resorption by osteoclasts. Moreover, our data is consistent with *in vivo* studies on Rac GTPases in osteoclasts (Croke et al., 2011; Vives et al., 2011; Zhu et al., 2016), suggesting that ELMO1/DOCK/Rac1 signaling nexus may play a crucial role in bone resorption by regulating the differentiation and function of osteoclasts.

The findings reported herein show that ELMO1 functions in the differentiation of osteoclasts and regulates their bone resorption activity *in vitro* and *in vivo*. It should be noted, however, that our results do not exclude the possible involvement of additional mechanisms of ELMO1 involving, for examples, osteoblasts and fibroblast-like synoviocytes that play vital roles in balance of bone metabolism. Further studies would shed light on the functions and mechanisms of ELMO1 in regulating bone resorption, which may lead to new therapeutic strategies for the treatment of diseases related to metabolic bone disorders.

## Methods

### Mice

ELMO1 deficient mice (Elmo1^–/–^) were purchased from European Mouse Mutant Archive. Mice were housed and bred under specific pathogen-free conditions in individual ventilated cages. Age- matched pairs of mice with normal physical characteristics were used in experiments (Supplementary Figure 2). All animal experiments were performed in accordance with the guidelines and regulations of School of Life Sciences, Shanghai University. Experimental protocols or methods were reviewed and approved by Ethics Committee of Shanghai University.

### Preparation of Bone Marrow-Derived Osteoclast Precursors

Bone marrow monocytes were prepared as osteoclast precursors for osteoclastogenesis assay *in vitro*. Under sterile conditions, the bone marrow cells from the tibias and femurs of 6– 8-week-old mice were flushed from the bone marrow cavity with Dulbecco’s modified Eagle’s medium (DMEM) (Hyclone) containing 10% fetal bovine serum (FBS) (Biological Industries), 100 U/ml penicillin and 100 μg/ml streptomycin. The cells were harvested by centrifugation at 500 g at room temperature for 5 min, then resuspended in 1 ml red blood cell lysis buffer and incubated for 1 min to remove the red blood cells. The clarified cells were cultured in DMEM containing 10% FBS, 1% penicillin and streptomycin in the presence of 50 ng/ml M-CSF (PeproTech).

### *In vitro* Osteoclast Differentiation and TRAP Staining

Osteoclast precursors obtained from BMMs were loaded into a 24-well plate at a density of 5 × 10^4^ cells/well and treated with 30 ng/ml RANKL (R&D Systems) in DMEM supplemented with 10% FBS, 1% penicillin and streptomycin and 50 ng/ml M-CSF for 4 days. The culture medium was replaced every 2 days. After osteoclast differentiation, the cells were fixed with 4% paraformaldehyde for 10 min and then stained for TRAP according to the manufacturer’s instructions of Acid Phosphatase Kit (Sigma-Aldrich). The TRAP- positive cells containing three or more nuclei were recorded.

### Immunofluorescence and Phalloidin Staining Assay

Osteoclast precursors were cultured in fibrinogen–coated glass-bottom dishes (NEST) at a density of 2 × 10^5^ cells per dish in the presence of RANKL (30 ng/ml). After 4 days, the cells were fixed with 4% PFA for 10 min and permeabilized with 0.1% TritonX-100, washed with phosphate buffer, and incubated with antibodies Rabbit anti-integrin β3 (Proteintech), anti-cadherins (ABclonal) for 2 h at 37°C with 100-fold dilution. Cells were then washed and incubated with secondary antibody Alexa Fluor 488 donkey anti-Rabbit H&G (Abcam) with 500-fold dilution. For phalloidin staining, the cells were incubated with Alexa Fluor 633-phalloidin (Life technologies) for 30 min followed by 4, 6-diamidino-2-phenylindole (DAPI) (Life technologies) staining for 2 min. The cells were imaged using a Zeiss 710 LSM confocal microscope (Zeiss, Germany).

### Quantitative Real-Time PCR (qRT-PCR)

Total RNA from osteoclasts that differentiated from BMMs was isolated with TRIzol reagent (Life technologies) according to the manufacturer’s instructions. Reverse transcription was performed with 2 μg of total RNA using FastQuant RT Kit (TIANGEN) and the resulting cDNAs were analyzed by real-time PCR for TRAP, NFATc1, DC-STAMP, integrin β3, cadherin-2, cadherin-11, and 18s RNA with the Power SYBR Green PCR Master Mix (Life technologies). 18s RNA was used as the invariant control. The relative mRNA level of target gene was expressed as 2^–△^^*Ct*^, in which △Ct are defined as the mean threshold cycle differences after normalization to endogenous control of 18s RNA. The primers used for qRT-PCR were as follows.

TRAP sense: 5′-CGACCATTGTTAGCCACATACG-3′;
TRAP antisense: 5′-TCGTCCTGAAGATACTGCAGG TT-3′;
NFATc1 sense: 5′- CCTGGAGATCCCGTTGC-3′;
NFATc1 antisense: 5′-GGTGTTCTTCCTCCCGATGT-3′;
DC-STAMP sense: 5′-AAAACCCTTGGGCTGTTCTT-3′;
DC-STAMP antisense: 5′-CTTCGCATGCAGGTA TTCAA-3′;
Integrin β3 sense: 5′-GCCTTCGTGGACAAGCCTGT-3′;
Integrin β3 antisense: 5′-GGACAATGCCTGCCAGTCTT-3′;
Cadherin-2 sense: 5′-AAGAGCGCCAAGCCAAGCAG-3′;
Cadherin-2 antisense: 5′-GGTACTGTGGCTCAGCA TG-3′;
Cadherin-11 sense: 5′-CTGGGTCTGGAACCAAT TCTTT-3′;
Cadherin-11 antisense: 5′-GCCTGAGCCATCAG TGTGTA-3′;
18s RNA sense: 5′-AGGCCCTGTAATTGGAATGA GTC-3′;
18s RNA antisense: 5′-GCTCCCAAGATCCAAC TACGAG-3′;

### Transwell Migration Assay

Osteoclast precursors migration assay was performed in a 24-well cell culture insert companion plate with 8-μm pore-sized Transwell filter (BD Falcon). 5 × 10^4^ cells suspended in 200 μl DMEM were added in triplicate to the upper chamber of the inserts, of which the membranes were pre-coated with 25 μg/ml fibronectin (Sigma-Aldrich). Then, 600 μl DMEM containing 50 ng/ml M-CSF, was added to the lower chamber for cell migration. After 24 h incubation, 200 μl 0.25% trypsin was added to the lower chamber to digest the cells for 5 min. Then the cells in lower chamber were collected and counted by flow cytometry. The migration rate was defined as the ratio of (experimental group-control group)/control group.

### Adhesion Assay

Osteoclast precursors adhesion assay was conducted in a 96-well plate. Cells suspended in 100 μl cell culture medium were seeded triplicate at a density of 1 × 10^5^ cells per well and incubated at 37°C for 60 min. Cells were washed with PBS and fixed with 4% PFA for 10 min after the non-adherent cells were aspirated out. The fixed cells were stained with crystal violet for 10 min and dissolved by DMSO for measuring absorbance at 570 nm using a microplate spectrophotometer.

### Analysis of RANKL-Induced Apoptosis

Osteoclast precursors were plated in 6-well plates and cultured in the presence of M-CSF and RANKL for 4 days. At the end, cells were collected and washed twice with PBS for analysis of RANKL-induced apoptosis using Annexin V/PI double staining kit (BD Pharmingen) following the manufacturer’s instructions. The apoptosis rate was determined by flow cytometry with Beckman CytoFLEX.

### Rac Activity Assay

Activation of Rac1 in response to RANKL was examined using Rac1 activation assay kit (Cytoskeleton). In brief, osteoclast precursors seeded on 6-well plates were stimulated with 30 ng/ml RANKL for the indicated time after starvation of serum for 2 h, then lysed in 200 μl ice-cold lysis buffer containing protease inhibitor cocktail. Cell lysates were clarified by centrifugation at 12,000 g for 1 min and 15 μl of these lysates was saved to detect the total level of Rac1, while the residual lysates were incubated with 10 μg of PAK-PBD beads at 4°C for 1 h. The beads were pelleted by centrifugation at 5,000 g at 4°C for 3 min, washed with wash buffer, and suspended with Laemmli sample buffer. The samples were subjected to SDS-PAGE and western blot analysis with an anti-Rac1 antibody.

### Activation of MAPKs and AKT

Osteoclast precursors were seeded into 6-well plates, stimulated with RANKL (30 ng/ml) for the indicated time and terminated directly by adding Laemmli sample buffer. Protein samples were separated by 10% SDS-polyacrylamide gel electrophoresis after being boiled for 10 min and then transferred to a polyvinylidene difluoride (PVDF) membrane. The PVDF membrane was blocked with 5% skim milk and probed with antibodies against p-JNK, p-p38, p-ERK, p-AKT, JNK, p38, ERK, and AKT (Cell Signaling Technology), Elmo1 (Abcam), and GAPDH (Tianjin Sungene Biotech) for indicated proteins.

### Generation of Rheumatoid Arthritis Mouse Model

RA mouse model was generated by serum transfer obtained from K/BxN mice according to the protocol as described (Pettit et al., 2001). In brief, arthritis of recipient mice was induced by caudal vein injection (10 μl serum/g weight of mouse) with K/BxN serum at days 0, 2, 7, and 12, and monitored throughout the next 21 days, whereas PBS was administered to control animals. Clinical index and ankle thickness were determined and measured at the indicated days.

### Microcomputed Tomography (Micro-CT) and Histology

Inflamed ankle joints were assessed by micro-CT and histology. For micro-CT imaging, ankle joints were analyzed using SkyScan1176 (Bruker). For each sample, about 200 slices with thickness of 18 μm were acquired to cover the entire width of the ankle. Images were reconstructed with the software of NRecon (Version: 1.6.9.8, Bruker) in 1,612 × 1,070 pixel matrices to provide a nominal resolution of 18 μm. 2-D slices from the 3-D stack of micro-CT images were evaluated for bone erosion. For histological analysis, ankle joints were wiped off skin and outer muscle, decalcified with 14% EDTA for 3 weeks, and then followed by paraffin embedding. Sections with thickness of 4 μm were cut for H&E and TRAP staining. The stained sections were observed under a Zeiss Axio Vert A1 microscope and the TRAP-positive cells were counted. Histopathological scoring was executed as described (Pettit et al., 2001).

### Pit Formation Assay

Osteoclast precursors from BMMs were cultured on Osteo assay plates (Corning) in the presence of M-CSF (50 ng/ml) and RANKL (30 ng/ml) for 4 days. Attached cells were removed and resorption lacunae were visualized by a Zeiss Axio Vert A1 microscope and quantified using ImageJ.

### Statistical Analysis

Statistical significance was evaluated with GraphPad Prism 6 using unpaired Student’s two-tailed *t*-test analysis of variance. *P* < 0.05 was considered significant.

## Data Availability Statement

The original contributions presented in the study are included in the article/Supplementary Material, further inquiries can be directed to the corresponding author/s.

## Ethics Statement

The animal study was reviewed and approved by the Minghong Wu, Ethics Committee of Shanghai University.

## Author Contributions

XC and JY contributed to conception and design of the study and wrote the manuscript. XL, YH, SY, YZ, and LH performed the experiments. XL and YH performed the statistical analysis. All authors contributed to manuscript revision, read, and approved the submitted version.

## Conflict of Interest

The authors declare that the research was conducted in the absence of any commercial or financial relationships that could be construed as a potential conflict of interest.

## Publisher’s Note

All claims expressed in this article are solely those of the authors and do not necessarily represent those of their affiliated organizations, or those of the publisher, the editors and the reviewers. Any product that may be evaluated in this article, or claim that may be made by its manufacturer, is not guaranteed or endorsed by the publisher.
