# Supplementary material for: ELMO1 Regulates RANKL-Stimulated Differentiation and Bone Resorption of Osteoclasts
Source: Front Cell Dev Biol. 2021 Jul 26;9:702916. doi: 10.3389/fcell.2021.702916 (PMC8350380; doi:10.3389/fcell.2021.702916)
Supplement: Supplementary file 1 [file Data_Sheet_1.PDF]

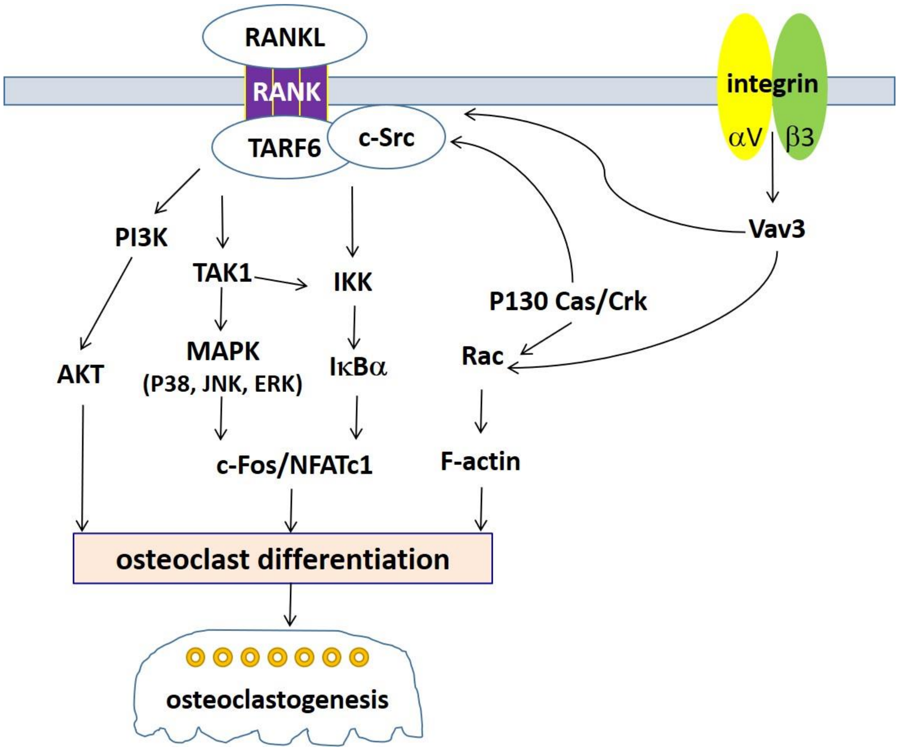

Figure S1. Signaling pathways in osteoclast differentiation.

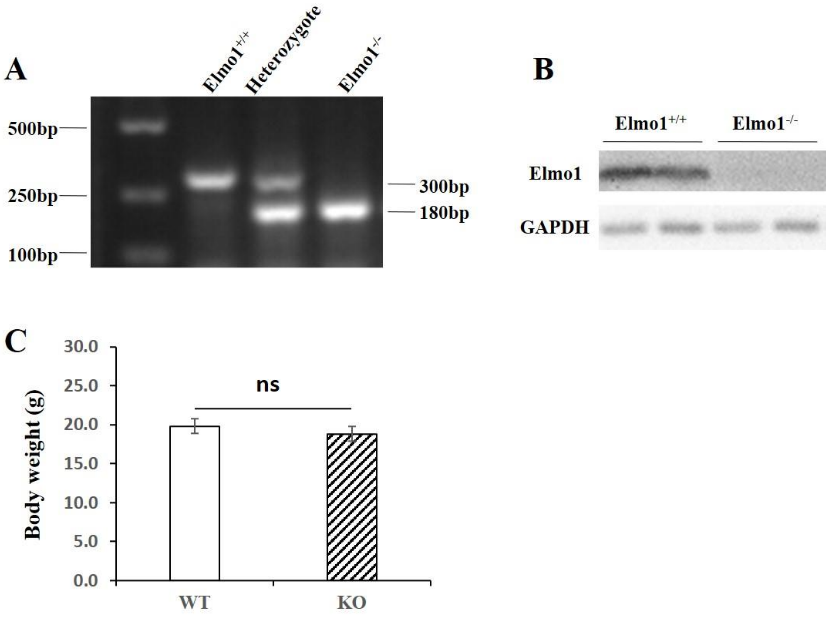

Figure S2. (A) Genotyping of offspring mice. Heterozygote mice (Elmo1+/-) were mated and PCR amplification of mouse tail DNA was used to identify the mice. Elmo1+/+ was detected with a product of 300 bp and Elmo1-/- with 180 bp. (B) Expression of Elm o1 in BMMs. Cell lysates were subjected to SDS-PAGE and immunoblot using specific antibodies against Elmo1 and GAPDH. (C) Body weight of mice aged 6–8 weeks (n=6). ns: not significant.

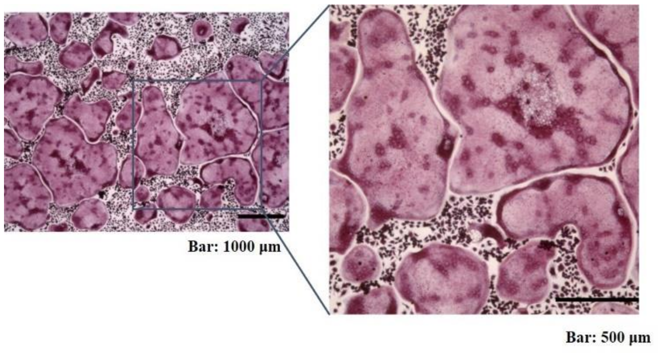

Figure S3. High magnification image of RANKL-induced osteoclast differentiation of BMMs.

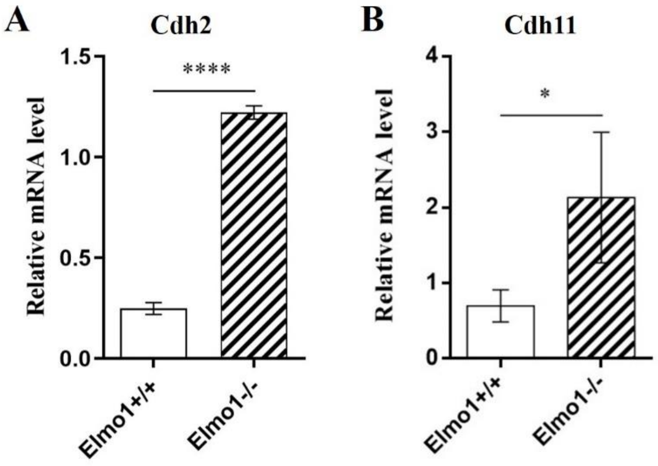

Figure S4. Relative mRNA level of cadherins in osteoclasts. Data of Cdh2 (n=3) and Cdh11 (n=4) are indicated as means ± SEM. Statistical significance was assessed by t-test, *P < 0.05, ***P < 0.001.

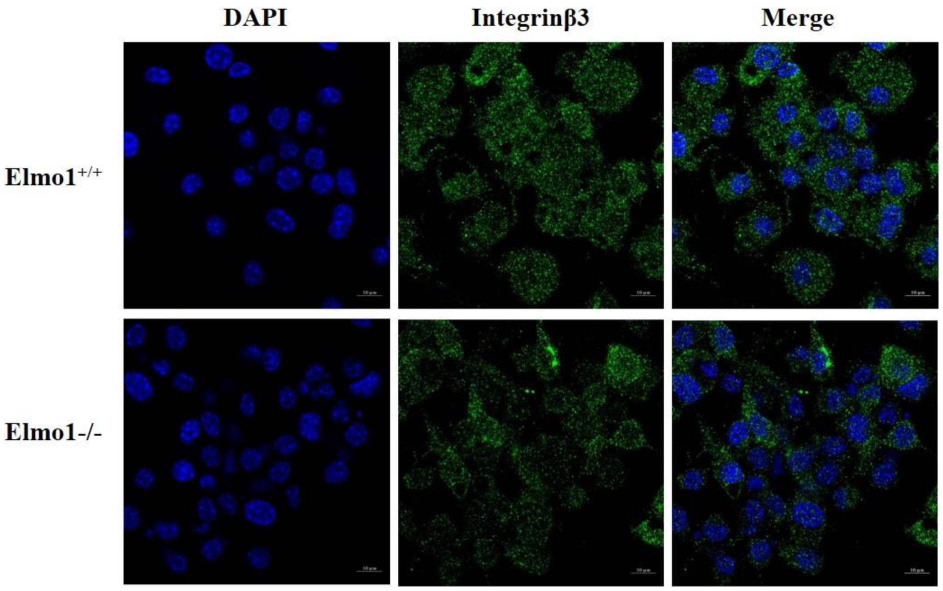

Figure S5. Immunostaining of the osteoclasts for integrin β3.

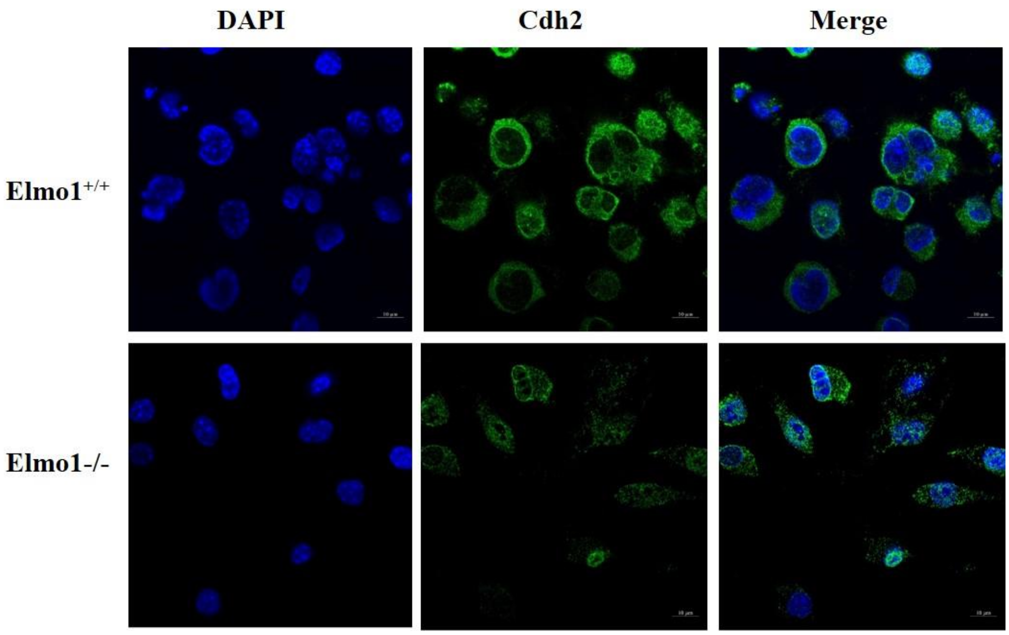

Figure S6. Immunostaining of the osteoclasts for Cdh2

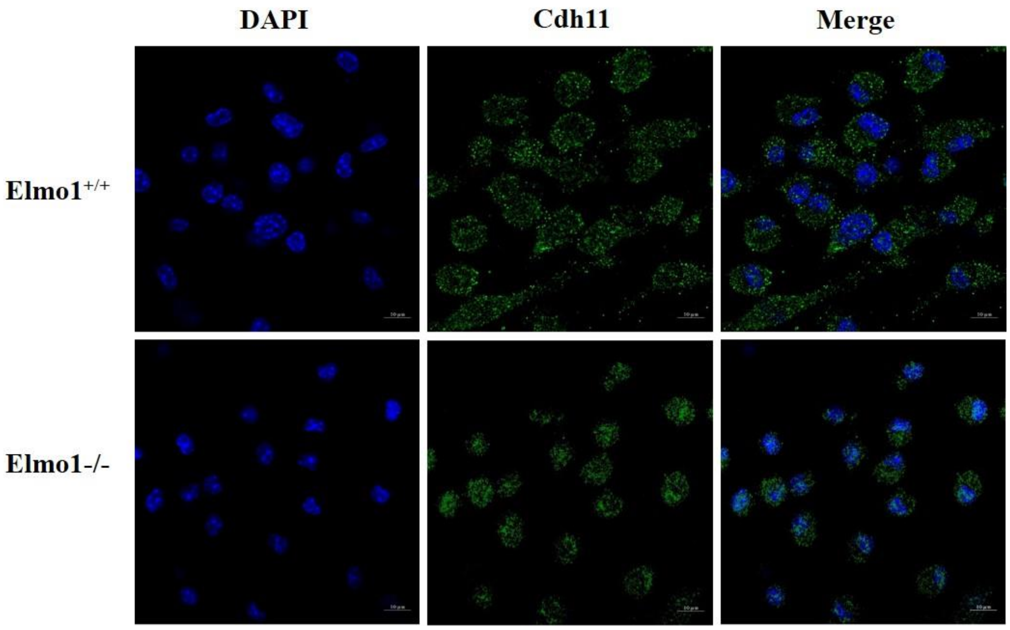

Figure S7. Immunostaining of the osteoclasts for Cdh11.

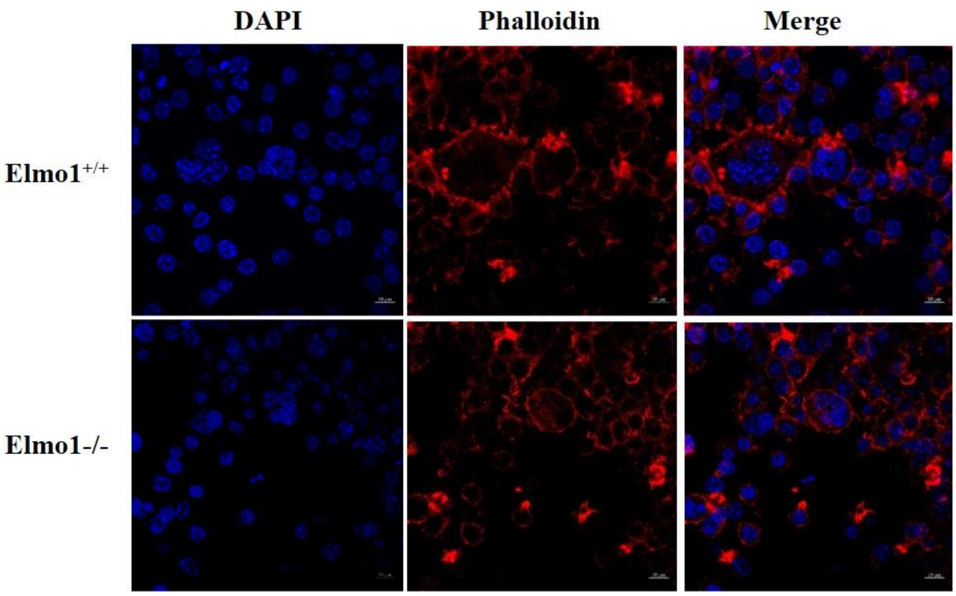

Figure S8. Staining F-Actin with Alexa Fluor 633-Phalloidin.

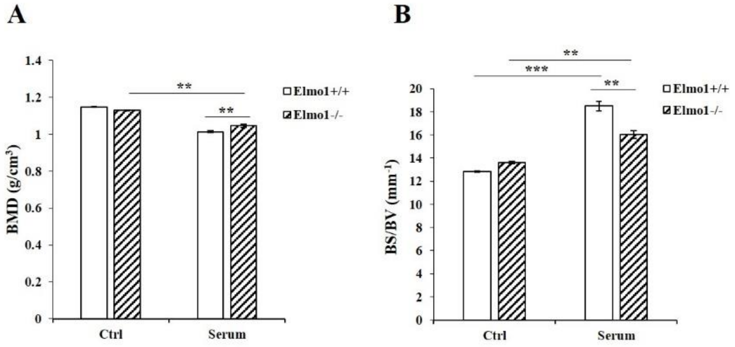

Figure S9. Bone erosion of ankle joints in WT and Elmo1 KO mice. (A) BMD, bone mineral density; (B) BS/TV, bone surface per total bone volume. Data are indicated as means ± SEM (n=6). Statistical significance was assessed by t-test, $^{**}P < 0.01$, $^{***}P < 0.001$.

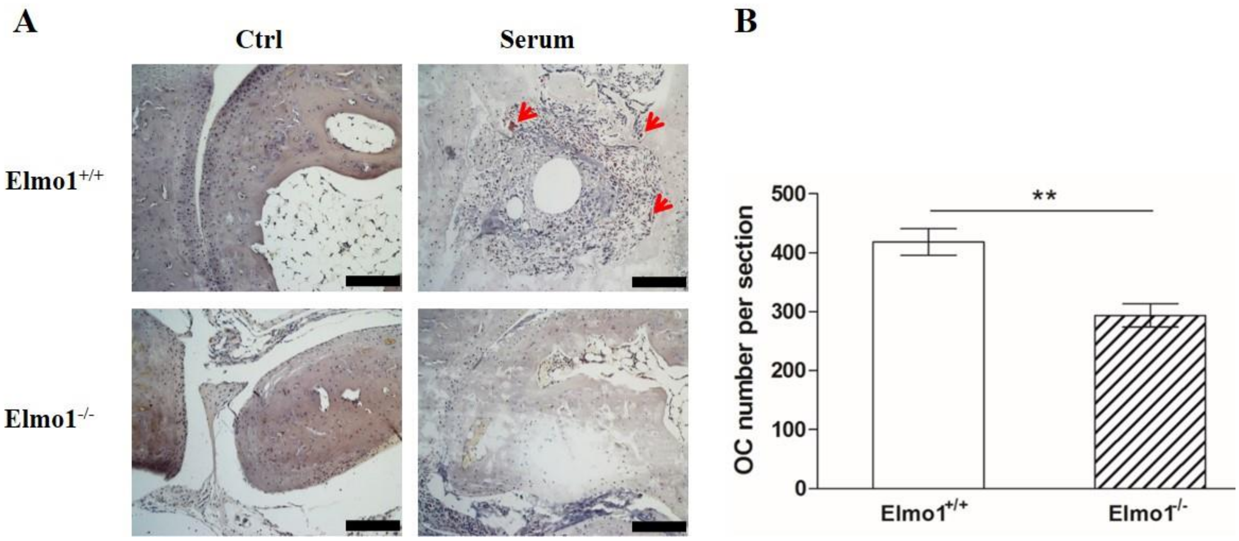

Figure S10. TRAP-stained corresponding histological joint sections for Figure 4E. (A) One representative picture is shown. Red arrows indicate osteoclasts. Scale bar, 100 μm. (B) Quantitative assessment of osteoclast numbers in ankle joint sections. Data are indicated as means ± SEM (n=5). Statistical significance was assessed by t-test, $^{**}P < 0.01$.
